# STMC: Statistical Model Checker with Stratified and Antithetic Sampling

**DOI:** 10.1007/978-3-030-53291-8_23

**Published:** 2020-06-16

**Authors:** Nima Roohi, Yu Wang, Matthew West, Geir E. Dullerud, Mahesh Viswanathan

**Affiliations:** 8 Microsoft Research Lab, Redmond, WA USA; 9 University of Southern California, Los Angeles, CA USA; 10 University of California, San Diego, USA; 11 Duke University, Durham, USA; 12 University of Illinois at Urbana-Champaign, Urbana, USA

## Abstract

is a statistical model checker that uses antithetic and stratified sampling techniques to reduce the number of samples and, hence, the amount of time required before making a decision. The tool is capable of statistically verifying any *black-box* probabilistic system that  can simulate, against probabilistic bounds on any property that  can evaluate over individual executions of the system. We have evaluated our tool on many examples and compared it with both symbolic and statistical algorithms. When the number of strata is large, our algorithms reduced the number of samples more than 3 times on average. Furthermore, being a statistical model checker makes  able to verify models that are well beyond the reach of current symbolic model checkers. On large systems (up to $10^{14}$ states)  was able to check 100% of benchmark systems, compared to existing symbolic methods in  , which only succeeded on 13% of systems. The tool, installation instructions, benchmarks, and scripts for running the benchmarks are all available online as open source.

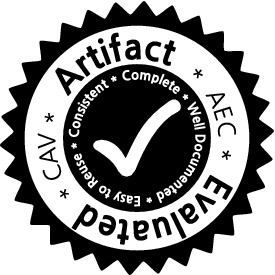



## Introduction

Statistical model checking (SMC) plays an important role in verifying probabilistic temporal logics on cyber-physical systems [1, 14, 15]. In SMC, we treat the objective bounded temporal specifications as statistical hypothesis, and infer their correctness with high confidence from samples of the systems. Compared to analytic approaches, statistical model checkers rely only on samples from the systems, and hence are more scalable to large real-world problems with complicated stochastic behavior [3, 6, 18].

To our knowledge, all existing SMC tools use independent samples. Admittedly, independent sampling is easy to implement, and it is the only option when the model is completely unknown. However, as shown recently in [24, 25], if the model is partially known, then we can exploit this knowledge to generate semantically negatively correlated samples to increase the sample efficiency in SMC. In [24, 25], we present the *stratified and antithetic sampling* techniques for discrete-time Markov chains (DTMC). In this work, we extend the technique to continuous-time Markov chains (CTMC), and implement the corresponding SMC algorithms in the tool  . The tool is evaluated on several case studies under hundreds of different scenarios, some of which are well beyond the capabilities of current symbolic model checkers. The results show that the sample efficiency can be significantly improved by using semantically negatively correlated sampling, instead of independent sampling.

This work also provides experimental comparisons between our SMC method and common symbolic model checking methods. Since we use large values for parameters in our case studies, it is no surprise that symbolic engines fail on many of them. However, without our results, the meaning of the word “large” is unclear. Our results give a good understanding of what is currently beyond the capabilities of symbolic engines in a popular tool like  . Next, restricting our attention to the cases in which symbolic engines successfully terminate, our results give us a helpful comparison between symbolic and statistical verification times. It is well-known that symbolic algorithms do not scale well, while statistical ones do. However, that knowledge alone does not give us any insight into how much more or less time a symbolic method requires compared to a statistical one. Finally, when a symbolic method terminates, one might argue that its result is far more valuable than the result of a statistical approach since statistical methods can produce incorrect results. Unfortunately, that is not entirely true. Since the complexity of solving a problem is too high in practice, many symbolic algorithms, including those in  , employ an iterative method to approximate probabilities. This approximation can be far from the actual probability, leading to incorrect model checking results (*e.g.,* [5]).

*Related Work.* Among the existing statistical model checkers,  [4, 12], MRMC [10], VESTA [19], YMER [27], and COSMOS [2] only support independent sampling on DTMC, CTMC, or other more general probabilistic models. PLASMA [9] also supports importance sampling. In importance sampling, although samples may have different weights, they are still generated independently. To our knowledge, our tool  is the only existing statistical model checker that employs semantically negatively related sampling on DTMC and CTMC.

## Stratified and Antithetic Sampling

**Fig. 1.**
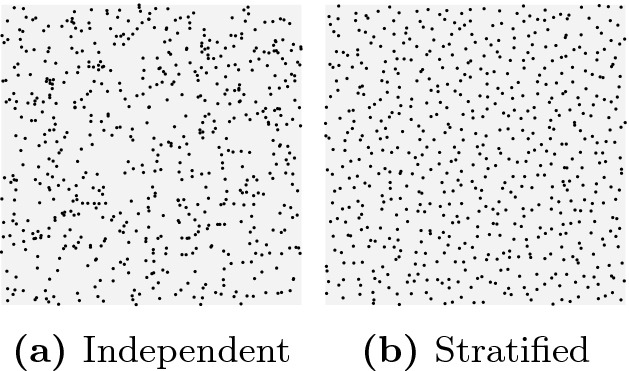
Uniform distribution

**Fig. 2.**
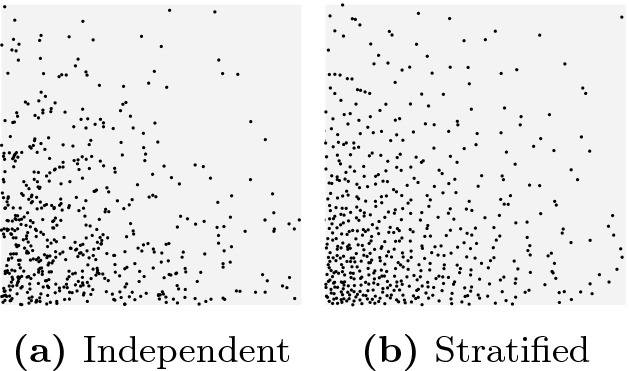
Exponential distribution

Stratified and antithetic samplings are two approaches for generating negatively correlated random samples. When using stratified sampling to draw *n* samples from a distribution, we divide the support into sets with equal measure, and then draw one sample from each partition. When using antithetic sampling, a random seed is first drawn from $x \in [0,1]$, and then two correlated samples are generated using *x* and $1-x$, respectively. Figures 1 and 2 compare independent and stratified sampling for 625 samples that we drew from the joint distribution of two random variables. In Fig. 1, each variable is uniformly distributed in [0, 1], and in Fig. 2, each variable is exponentially distributed with rate 3 (we only show samples that are within the unit square). It is clear that the stratified samples are (visually) better distributed in both figures.

We have shown in [24] that by choosing a proper representation of a Markov chain, the stratified sampling technique can be applied to generate semantically negatively correlated sample paths. This technique reduces the sampling cost for statistically verifying temporal formulas. In the rest of this section, we list two algorithms: Stratified sampling of a CTMC, and stratified sequential probability ratio test for a CTMC. The antithetic variants are simpler and we do not present them here for the lack of space. Compared to our algorithms in [24], there are two main differences. First, we present these algorithms for CTMCs instead of DTMCs, as they are slightly more involved. Second, for the stratified sampling of a CTMC, our algorithm supports stratification over multiple steps directly.

Algorithm 1 shows the pseudo-code for stratified sampling of a CTMC; to obtain a stratified sampling algorithm for DTMC, we only need to remove  ,  ,  ,  ,  , and  . It takes two inputs: $\psi$, a temporal formula that we want to evaluate on every sampled path, and  , the number of strata at every step. This is a non-empty list of positive integers. Let *K* be the length of this list, and *N* be the product of its elements. If the $i_\mathrm{th}$ item of the list is *n* then the number of strata at steps $i,i+K,i+2K,i+3K,\ldots$ must be *n*.^1^ The algorithm simultaneously simulates *N* paths and terminates after the value of $\psi$ on all these paths are known. Inside the main loop, simulation is performed incrementally, *K* steps at a time. Random permutations  ,  , and variables  ,  are used to make simulations of every *K* steps and random numbers  and  (defined later in the code) independent of each other. The number of strata at every step is an input to this algorithm. Using that number, variables  and  determine which strata we should use at step *s*. Finally,  is a uniformly distributed stratified sample in [0, 1). However, we need an exponentially distributed stratified sample, which is precisely what  gives us.

Algorithm 2 shows pseudo-code for statistical verification of CTMC and DTMC using stratified samples. The algorithm is quite simple. It keeps sampling using Algorithm 1 and computes the average and variance of the values it receives until a termination condition is satisfied. Checking the termination conditions after every step suggests using an online algorithm for computing the mean and variance of samples. We use Welford’s online algorithm [26] in our implementation.

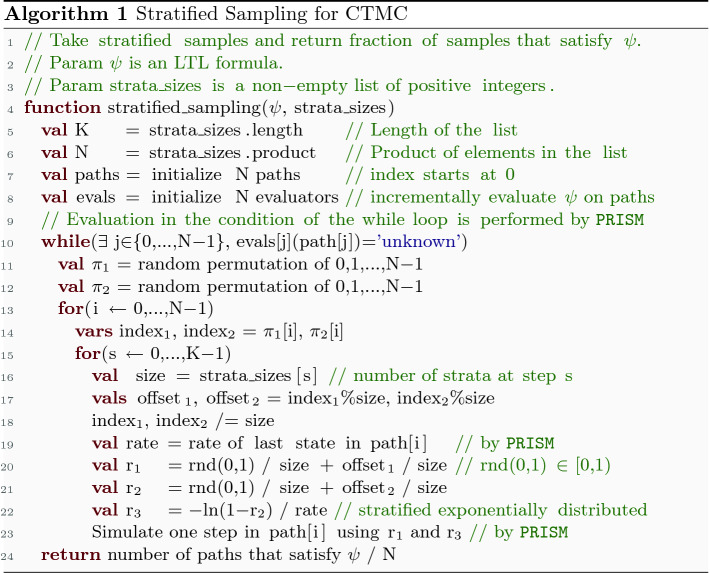



Finally, one can extend the following results from [24] to include CTMC.

### Theorem 1

Let $\psi$ be a bounded LTL formula.

1. The output of Algorithm 1 has the same expected value as the probability of a random path satisfying $\psi$.
2. If $\psi$ is of the form $\psi _1\mathcal {U}_{I}\psi _2$, such that the set of states satisfying $\psi _2$ is a subset of the same set for $\psi _1$, then the satisfaction values of different paths simulated by Algorithm 1 are non-positively correlated.

### Theorem 2

The sampling cost of Algorithm 2 is asymptotically no more than the sampling cost of SPRT [20] using i.i.d. samples.

## Tool Architecture

**Fig. 3.**
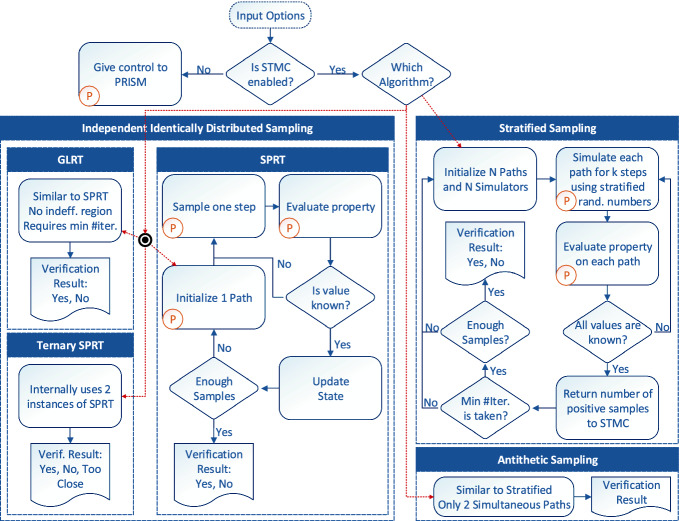
Architecture of  . Boxes marked with letter ‘P’ use  directly. *N* is the number of strata, *K* is the length of strata-size list (see option strata_size below).

We have implemented our algorithms in  and published it under the GNU General Public License v3.0. The tool can be downloaded from https://github.com/nima-roohi/STMC/, where installation instructions, benchmarks, and scripts for running the benchmarks are located. We use  to load models from files, simulate them, and evaluate simulated paths against non-probabilistic bounded temporal properties. Therefore,  is capable of statistically verifying any model, as long as it can be simulated by  , and bounded temporal properties can be evaluated on single executions of that model. Figure 3 shows  at a very high level. Boxes marked with ‘P’ are where we directly use  .

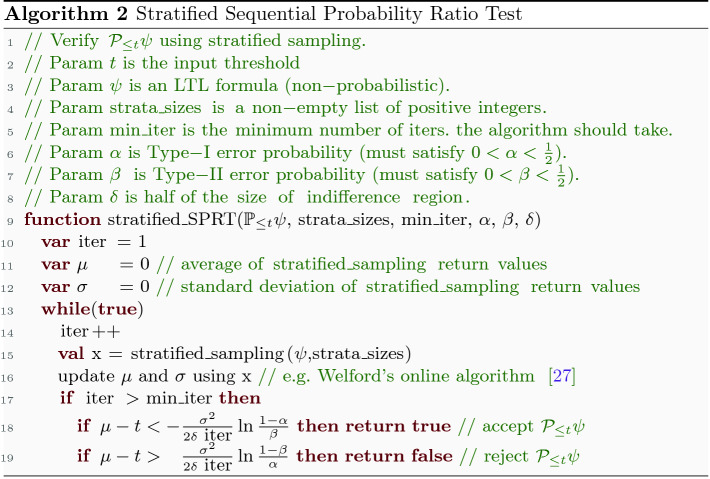



Executions of  are configured through different options/switches. The most basic options are help, which prints out a list of switches for both  and  , and stmc, which enables the tool (without stmc, everything will be passed to  , pretty much like  was not there in the first place). Statistical verification is enabled using option sim; it is always required when stmc is used. The sampling method is specified using option smp_method or sm. Possible values for the sampling method are independent, antithetic, and stratified. Using option hyp_test_method or hm, users also have to specify a hypothesis testing method that they would like to use. Supported values for this option are currently SPRT, TSPRT, GLRT, and SSPRT. SPRT is used for the sequential probability ratio test [20]. This algorithm has already been implemented in  and in our experience it has a very similar performance to our implementation (SPRT in Sect. 4 refers to the implementation from  ). We use our implementation for the next option, TSPRT. Sequential probability ratio test assumes that the actual probability is not within the $\delta$-neighborhood of the input threshold. If this assumption is not satisfied, then the algorithm does not guarantee any error probability. TSPRT, which stands for Ternary SPRT, solves this problem by introducing a third possible answer: TOO_CLOSE. The algorithm was introduced in [28]. *Without* assuming that the actual probability is not within the $\delta$-neighborhood of the input threshold, TSPRT guarantees Type-I and Type-II error probabilities are bounded by the input parameters $\alpha$ and $\beta$, respectively. Furthermore, it guarantees that if the actual probability and the input threshold are not $\delta$-close, then the probability of returning TOO_CLOSE is less than another input parameter $\gamma$; we call this Type-III error probability. The sequential probability ratio test was originally developed for simple hypotheses, and the test is not necessarily optimal when composite hypotheses are used [13]. To overcome this problem, the generalized likelihood ratio test (GLRT) was designed in [7]. The algorithm does not require an indifference region as an input parameter and provides guarantees on Type-I and Type-II error probabilities *asymptotically*. The main issue with this test is that since probabilistic error guarantees are asymptotic, for the test to perform reasonably well in practice (*i.e.*, respect the input error parameters), a correct minimum number of samples must be given as an extra input parameter. If this parameter is too large then the number of samples will be unnecessarily high, and if the parameter is too small then the actual error probability of the algorithm could be close to 0.5, even though the input error parameters are set to, for example, $10^{-7}$. The last possible value for hyp_test_method is SSPRT, which stands for Stratified SPRT. This option is used whenever stratified or antithetic samplings are desired.

When stratification is used, the number of strata should be specified using option strata_size or ss. It is a comma-separated list of positive integers. For example, 4, 4, 4, 4, 4, 4 specifies 4 strata for six consecutive steps (4096 total), and 4096 specifies 4096 strata for every single step. Note that in both of these examples, stratified sampling simultaneously takes 4096 sample paths, which requires more memory. However, we saw in our experiments that for non-nested temporal formulas, at most two states of each path are stored into memory. Therefore, even larger strata sizes should be possible. This was the most challenging part of the implementation, because the simulator engine in  is written assuming that paths are sampled one by one. However, if we followed the same approach in  , we would have to store every random number that was previously generated, which increased the amount of memory used for simulation from $\mathcal {O}(1)$ to $\mathcal {O}(N\times L)$, where *N* is the number of strata and *L* is the maximum length of simulated paths. By simulating the paths simultaneously, we only use $\mathcal {O}(N)$ bytes of memory. Next, Type-I, Type-II, Type-III, and half of the size of the indifference region are specified using alpha, beta,^2^ gamma and delta, respectively (not every algorithm uses all of these parameters). Finally, most algorithms that use variance in their termination condition, require help when sample variance remains zero after the first few iterations.  uses min_iter for this purpose, and  uses simvar.

**Fig. 4.**
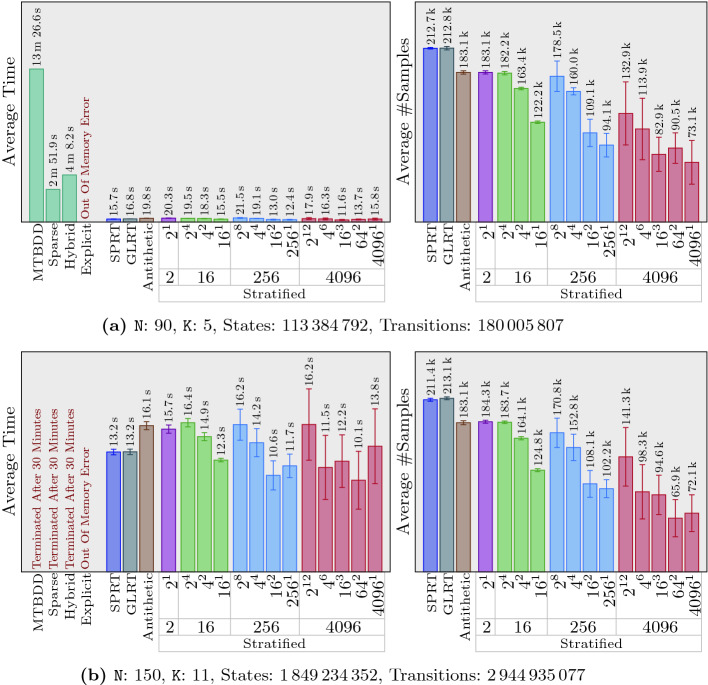
NAND multiplexing (DTMC - macOS) [17]

**Fig. 5.**
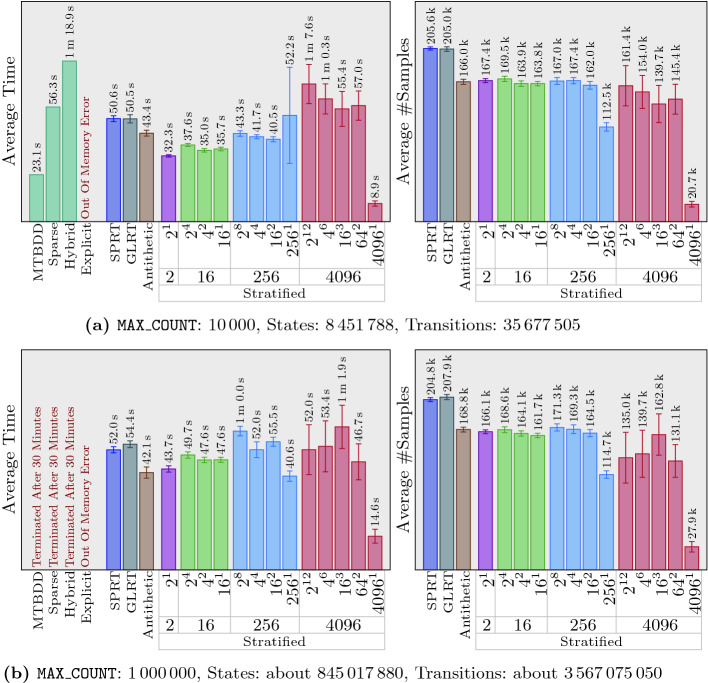
Embedded control system (CTMC - Ubuntu) [11, 16]

**Fig. 6.**
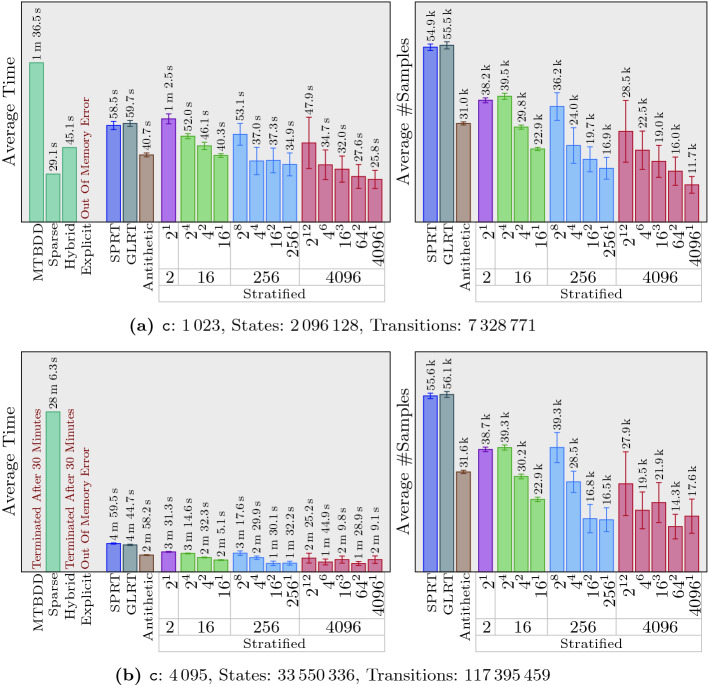
Tandem queueing network (CTMC - macOS) [8]

## Experimental Results

We evaluated our algorithms on 10 different sets of examples. Each set contains four variations of the same problem with varying parameters and, hence, various sizes, and each of those variations includes four symbolic tests as well as 16 statistical ones. Furthermore, we repeat each of the statistical tests 20 times, to compute 95% confidence intervals for time and number of samples taken by the statistical algorithms. This gives us a total of 800 tests and $12\,960$ runs to obtain results for those tests. Regarding the stratified sampling, for each variation, we consider 13 settings in 4 groups. Each group uses a different number of strata: 2, 16, 256, and 4096. When the number of strata is more than 2, we also consider different possibilities for how to divide strata among different steps. For example, when 256 strata are used, $256^1$ means every step has 256 strata, but different steps are independent of each other. On the other hand, $2^8$ means every step has only two strata, but stratification is performed over every 8 consecutive steps.

For the sake of space, we only present 15% of our results in this paper. Full experimental results are available at https://nima-roohi.github.io/STMC/#/benchmarks. Also, all the benchmark source files, along with scripts for running them, can be obtained from the tool’s repository page https://github.com/nima-roohi/STMC/. The parameters we chose resulted in large systems, and significant time has been spent to run and collect the results. To perform our experiments faster, we ran all of our tests using four processes (using option ‘-mt 4’). We also divided out our 10 sets of examples into two groups and ran each set on one of two machines. One of them is running Ubuntu 18.04 with an i7-8700 CPU 3.2 GHz and 16  GB memory, and the other one is running macOS Mojave with an i7 CPU 3.5 GHz and 32 GB memory.  ’s webpage contains a short description for each example and a link to another page for the full explanation. We end this section with a few notes regarding our results.

1. Like any statistical test that is run in a black-box setting, we need to assume simulation of every path will eventually terminate. In fact,  uses the parameter simpathlen, with $10\,000$ as its default value, to restrict the maximum number of simulation steps in each path. Currently, simpathlen can be as large as $2^{63}-1$, which is more than enough in most practical applications.
2. To make the configurations less in favor of statistical algorithms, we used small values for $\alpha$, $\beta$, and $\delta$ in our benchmarks (between 0.0001 and 0.001). Also, we have estimated the actual probabilities using a symbolic model checker or using a statistical algorithm in  and set the threshold close to the actual probability. These settings cause the statistical algorithms to take more samples, which indeed makes it possible for us to observe the effect of antithetic and stratification on the number of samples. As a side effect, we did not observe any performance benefits of GLRT over SPRT.
3. In many of our examples, the variance is particularly high when strata size is 4096. This is because in our benchmarks, whenever 4096 strata are used, we set the minimum number of iterations to 2 (*i.e.,* 8192 samples). This means that when the average number of samples in our results is, for example, around $20\,000$, only 5 iterations have been taken on average, and every iteration adds or removes about $20\%$ of the samples from the test.
4. In general, the more strata we use, the greater reduction in the number of samples we observe. Also, the performance of antithetic sampling is similar to the case of using only two strata. Our best results are obtained when $4096^1$ is used for the number of strata. For example, in Fig. 5a, comparing SPRT and $4096^1$ strata shows almost ten times reduction in the average number of samples. The tool’s webpage contains an example in which stratification reduces variance to 0. This results in the termination of the algorithm immediately after a minimum number of samples have been taken, giving us 3 orders of magnitude reduction in the number of samples.

## Conclusion

We presented our new tool called  for statistical model checking of discrete and continuous Markov chains. It uses antithetic and stratified sampling to improve the performance of a test. We evaluated our tool on hundreds of examples. Our experimental results show that our techniques can significantly reduce the number of samples and hence, the amount of time required for a test. For example, when $4096^1$ strata were used, our algorithms reduced the number of samples more than 3 times on average. We have implemented our tool in  , and published it online under GNU General Public License v3.0. We would like to extend  to support other stratification-based algorithms. In particular, stratified sampling in model checking Markov decision processes, and temporal properties that are defined on the sequence of distributions generated by different types of Markov chains (see [21–23] for examples).

## Footnotes

1 The current version of  only handles one initial state for simulation. Therefore, there will be no stratification for initializing paths.

2 To the best of our knowledge,  always assumes $\alpha =\beta$.
